# Achieving Nasal Septal Cartilage In Situ Regeneration: Focus on Cartilage Progenitor Cells

**DOI:** 10.3390/biom13091302

**Published:** 2023-08-25

**Authors:** Chong Zhang, Guanhuier Wang, Yang An

**Affiliations:** Department of Plastic Surgery, Peking University Third Hospital, 49 North Garden Road, Haidian District, Beijing 100191, China; 1810301339@pku.edu.cn (C.Z.);

**Keywords:** cartilage progenitor cell, cartilage tissue engineering, in situ regeneration, nasal cartilage, rhinoplasty

## Abstract

The nasal septal cartilage plays an important role in preventing the collapse of the nasal bones and maintaining the appearance of the nose. In the context of inherent difficulties regarding septal cartilage repair and the shortage of cartilage graft resources for regeneration, tissue engineering, especially the in situ strategy based on scaffolds, has become a new prospect and become one of the most promising approaches. Given that it is difficult for chondrocytes to achieve directional migration and secrete matrix components to participate in tissue repair after cartilage injury, cartilage progenitor cells (CPCs), with great migratory ability and stem cell characteristics, have caught the attention of researchers and brought hope for nasal septal cartilage in situ regeneration. In this review, we first summarized the distribution, characteristics, isolation, and culture methods of nasal septal CPCs. Subsequently, we described the roles of migratory CPCs in cartilage regeneration. Finally, we reviewed the existing studies on CPCs-based cartilage tissue engineering and summarized the strategies for promoting the migration and chondrogenesis of CPCs so as to provide ideas for achieving nasal septal cartilage in situ regeneration.

## 1. Introduction

The nasal septal cartilage plays an important role in preventing the collapse of the nasal bones and maintaining the appearance of the nose. Clinically, trauma, surgery, tumor, infection, and congenital factors may cause nasal septal deformity and defect, which affects the respiratory function of the patient’s nose and results in morphological deformity, impacting the patient’s daily life [1]. Therefore, severe cases often require surgical intervention and the implantation of new cartilage. Nasal septal cartilage offers easy accessibility compared to rib cartilage and articular cartilage and shows better tissue stability and higher chondrogenic capacity [2,3]. Therefore, nasal septal cartilage has become one of the important donor sites for autologous cartilage transplantation and is widely used, not only in plastic surgery, but also in joint surgery [3,4,5].

In the context of the inherent difficulties for septal cartilage self-repair and the shortage of cartilage graft resources for transplant surgery, tissue engineering strategies have revealed a new prospect and become one of the most promising approaches. In the study by Bermueller et al. [6], an orthotopic rat model for nasal septal repair was developed in which the marine collagen chondrocyte-seeded/unseeded scaffolds were evaluated. In similar rabbit models, elastin–gelatin–hyaluronic acid and decellularized porcine nasal septal cartilage scaffolds were also evaluated, both using auricular cartilage as seed cells [7,8]. However, traditional tissue engineering methods based on seed cells and scaffolds often required multiple steps, including tissue acquisition, seed cell isolation and culture, scaffold implantation, in vitro culture, and construct implantation. This causes patients to undergo at least two surgical procedures, with significant time spent in between, and to suffer from donor-site injury, accompanying pain, and risk of donor-site complications.

In situ regeneration is one of the most sought-after tissue engineering methods, requiring only the implantation of a scaffold and the recruitment of autologous in situ cells to achieve tissue regeneration. The absence of additional seed cells ensures biosafety and reduces the number of pre-implantation procedures, which facilitates clinical translation. The successful recruitment of autologous in situ cells is the key to in situ regeneration. However, it is difficult for chondrocytes harbored in the pyknotic cartilage matrix to achieve directed migration and secrete matrix components to achieve satisfying tissue repair after cartilage injury.

Cartilage progenitor cells (CPCs) were first reported to be found in bovine joints [9], and subsequently, nasal CPCs (NCPCs) were isolated and identified in human nasal septal cartilage in 2011 [10]. NCPCs were proven to exhibit the basic characteristics of stem cells, such as self-renewal, multidirectional differentiation potential, and clonal formation; more importantly, they also showed an even a stronger migratory capacity than bone marrow-derived mesenchymal stem cells (BMSCs) [11]. Therefore, these cells bring new hope for nasal septal cartilage in situ regeneration [10,11]. However, the natural recruitment of NCPCs is insufficient to achieve the complete repair of cartilage defects [12].Therefore, researchers hope to design scaffolds with high CPCs recruitment capabilities, or to screen and utilize biochemical additives such as chemokines, to effectively improve cartilage in situ regeneration.

**Figure 1:**
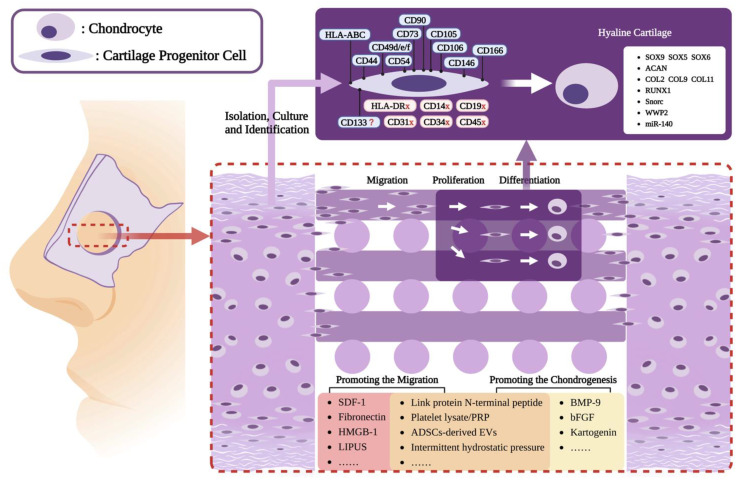
Achieving nasal septal cartilage in situ regeneration with a focus on CPCs: Facing difficulties for septal cartilage repair and the shortage of cartilage graft resources for regeneration, tissue engineering, especially the in-situ strategy based on scaffolds, has become one of the most promising approaches. CPCs, with great migratory ability and stem cell characteristics, have caught the attention of researchers and brought hope for nasal septal cartilage in situ regeneration. Strategies for promoting the migration and chondrogenesis of CPCs are constantly being improved to induce more CPCs to migrate to tissue engineering scaffolds and to subsequently differentiate in order to achieve satisfactory cartilage in situ regeneration. Created with BioRender.com (accessed on 9 April 2023).

This review is aimed at summarizing the existing basic information on NCPCs and existing CPC-related tissue engineering designs to provide support and references for the in situ regeneration of nasal septal cartilage (Figure 1). Specifically, we first summarized the distribution, isolation, and culture methods of NCPCs. Subsequently, we described the roles of migratory CPCs in cartilage regeneration. Finally, we reviewed the existing studies on CPCs-based cartilage tissue engineering and summarized the strategies for promoting the migration and chondrogenesis of CPCs to provide ideas for achieving nasal septal cartilage in situ regeneration.

## 2. Isolation and Characterization of CPCs

### 2.1. Histological Distribution and Characteristics of NCPCs

**Figure 2:**
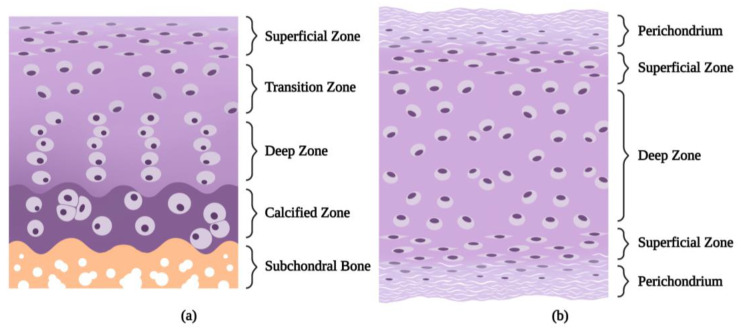
(**a**) The four-layer structure over the subchondral bone of the articular cartilage: the superficial zone, the transition zone, the deep zone, and the calcified zone. (**b**) The three-layer structure of the nasal cartilage and the perichondrium covering it. Created with BioRender.com (accessed on 15 December 2022).

Nasal cartilage mainly includes the central septal cartilage, two upper lateral cartilages, and two lower lateral cartilages. These cartilaginous structures are made up of hyaline cartilage, consisting of an extracellular matrix (ECM) and chondrocytes, and which was formerly considered to be the only existing cell component [13]. With the perichondrium on its surface and a sandwich-like structure, as opposed to a structure transiting into the subchondral bone, the nasal cartilage is obviously different from the articular cartilage (Figure 2). When approaching the deeper zone, the cells gradually become larger, more rounded, and less frequent [13]. The perichondrium is rich in type I collagen, thus distinguishing it from the cartilage tissue, which contains type II only [14]. The perichondrium, however, is not a membrane that can be easily separated from cartilage tissue. In previous histological studies, the demarcation between the perichondrium and the cartilage surface was not easily distinguished, but exhibited a more transitional evolution [14,15], and the demarcation between the cartilage and the perichondrium and between the perichondrium and the lamina propria was more regular in the posterior versus the anterior septum [16]. Therefore, it is difficult to distinguish the flat cells in the superficial layer of cartilage from those in the deep layer of the perichondrium. But it is confirmed that a subpopulation of CPCs exists among these flat cells, playing an important role in cartilage regeneration. Compared to the differentiated chondrocytes, in addition to the morphological differences, CPCs exhibit the basic characteristics of stem cells (such as self-renewal, multidirectional differentiation potential, and clonal formation) and the stem cell markers on their surfaces, along with a stronger migratory capacity [11].

Early in 1995, in the study of Pirsig et al. [17], an implant of demineralized bovine bone matrix enfolded in a pedicled flap of autogenic ear perichondrium was transformed into the autogenic cartilage of a boy’s nose. Tcacencu et al. reported that in the cricoid cartilage defect rabbit models, new cartilage originated from the mesenchymal progenitor cells of a host perichondrium adhered to cricoid cartilage by treatment with recombinant human bone morphogenetic protein-2 (BMP-2) [18]. In the study of Kaiser et al. [12], a 1.0 cm × 2.5 cm central basal segment of the rabbit’s septal cartilage was removed, and both sides of the septal mucosa were then reapproximated, resulting in the substantial regrowth of cartilage in the defect covered by the perichondrium.

Similar flat cells are also distributed on the surface of articular cartilage and other cartilage tissues. In early studies, researchers only speculated that there were progenitor cell subsets on the surface of the cartilage [19]. In 2004, Dowthwaite et al. isolated articular CPCs (ACPCs) from the surface zone of bovine articular cartilage and identified them adequately for the first time [9]. Later in 2011, Shafiee et al. first isolated and identified NCPCs in human nasal septal cartilage [10]. Currently, CPCs have been isolated from different species and cartilage tissue of different body organs. However, research on NCPCs is still limited, and only a few articles, which are summarized in the following table (Table 1), isolate human NCPCs and discuss their features and functions.

**Figure 3:**
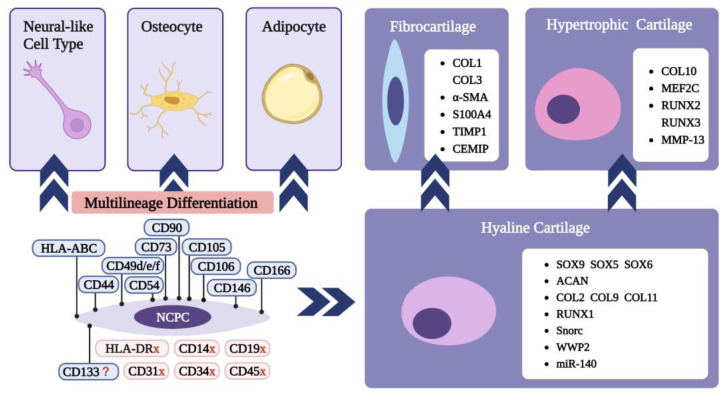
The surface markers on NCPCs and the multilineage differentiation of CPCs. Created with BioRender.com (accessed on 27 January 2023).

Like CPCs from other sites, NCPCs possessed properties of adult stem cells, such as self-renewal capacity, multilineage differentiation potential, and MSC-related surface markers (Figure 3) [25]. NCPCs possessed the ability to differentiate into adult chondrocytes without additional growth factors, which did not decrease with 10 passages [10,11,15,20,21,22,23,24]. Additionally, NCPCs were shown to differentiate into osteoblasts in the osteogenic-inducing medium [10,11,15,20,21,23,24]. However, the adipogenesis capacity of CPCs was controversial. In some studies, CPCs failed to differentiate into adipocytes and accumulate cytoplasmic lipid droplets [10,15,20], while possessing adipogenesis differentiation potential in others [11,23,24]. In addition, in the study of Shafiee et al. [10], CPCs possessed the capacity to differentiate into neural-like cell types, with positive expression of the mature neural markers b-tubulin III (b-TUB) and microtubule-associated protein-2 (MAP-2), nestin, and neural-specific enolase (NSE).

Summarizing the existing studies on NCPCs, these cells were positive for the surface markers CD44, CD49d/e/f, CD54, CD73, CD90, CD105, CD106, CD146 and CD166, and were negative for CD14, C19, CD31, CD34, and CD45 (Table 1). In the study by Shafiee et al. [10,21], the higher expression of CD133 was distinctly different in NCPCs compared with the other cell types, including adipose-derived stem cells (ADSCs), BMSCs, and ACPCs. However, in other studies, NCPCs were negative for CD133 [11,20]. Moreover, in the study by do Amaral et al. [15], NCPCs were negative for CD146, which was a distinct marker of CPCs in most studies.

### 2.2. The Characteristic-Based Isolation of CPCs

**Figure 4:**
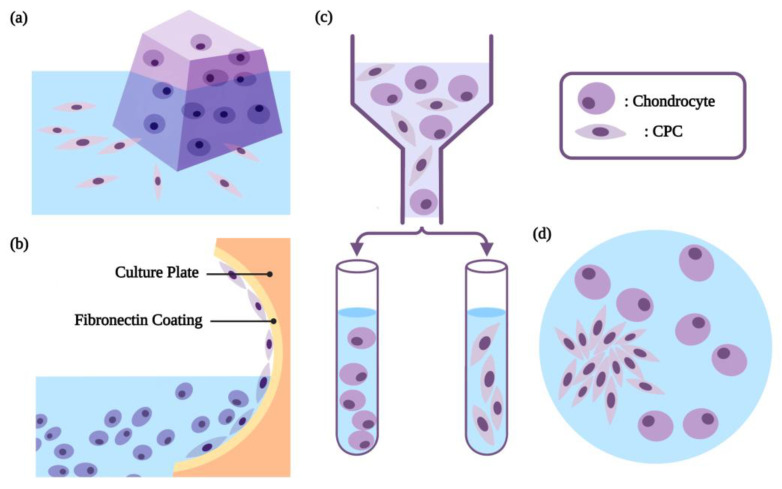
Different methods of CPCs isolation: (**a**) method of migration; (**b**) fibronectin adhesion assay; (**c**) cell sorting; (**d**) colony screening. Created with BioRender.com (accessed on 27 January 2023).

Different methods could be used to isolate CPCs, including fibronectin adhesion assay, method of migration, cell sorting, enzymatic digestion, and colony screening (Figure 4). Before the isolation, the cartilage tissue was usually washed with phosphate buffer saline (PBS) and cut into small 1 mm^2^ pieces. The most commonly used fibronectin adhesion assay protocol is based on the strong adhesion to fibronectin of the CPCs, with fibronectin receptors, integrin-α5 and integrin-β1, on their surface [9]. The cells derived from cartilage tissue by enzymatic hydrolysis were seeded on the fibronectin-coated plate for 20 min. Afterward, the nonadherent cells were removed, and the CPCs were harvested. This is an attractive method, as it is time-saving and stable, while pure chondrocytes can be simultaneously harvested. Moreover, with a strong migration ability, CPCs could migrate out of the cartilage tissue mass, with or without stimulation. Therefore, taking advantage of this property, CPCs could be isolated from the small pieces of cartilage tissue within 7–14 days of in vitro culture, with or without being triggered by cartilage injury [11,26]. Alternatively, CPCs could also be sorted from the total cell population via cell sorting methods like immunomagnetic separation or fluorescence-activated cell sorting (FACS), based on the specific expression of CPC surface markers, mainly CD105, CD146, and CD166, individually or in combination [27,28,29,30,31]. In addition, collagenase was used for isolation, combined with colony screening. Some articles used collagenase type I alone for isolation, and the obtained cells were usually derived from the perichondrium, which contains collagen type I; thus, it was worth investigating whether or not they were CPCs [6,15,22]. In the other studies, the cells digested by the enzyme were later cultured at a low density. While chondrocytes in culture rapidly lost their phenotype, CPCs expressed as high-efficiency colony-forming cells, with multi-differentiation potential [32]; thus, the forming colony could be harvested as CPCs [33].

However, CPCs isolated in different ways may possess different characteristics. In the study of Elizabeth et al. [34], CPCs isolated through migration (MCPCs) displayed lower levels of hypertrophy markers (RUNX2 and COL1A1), higher levels of chondrogenic markers (Aggrecan and COL2A1/COL1A1 ratio), and higher levels of GAG/DNA, with stronger staining. These results suggested that MCPCs showed a greater potential for cartilage regeneration, especially in situ tissue engineering, due to their basic migration features.

## 3. The Role of Migratory CPCs in Cartilage Regeneration

### 3.1. The Migrating CPCs in Cartilage

CPCs have been shown to play an important role in the process of chondrogenesis and regeneration. When we talk about the physiological and pathological functions of CPCs, their migration is often the focus of our attention.

#### 3.1.1. Migrating CPCs in the Injury Cartilage and Inflammatory Environment

After injury in the bovine osteochondral explant, CPCs migrated to the injury site in 7–14 days, via the increase in high mobility group box chromosomal protein 1 (HMGB-1) and the receptor for advanced glycation end products (RAGE)-mediated chemotaxis, and promoted the repair of chondral damage [26]. In the study of Wagner et al. [35], HMBG-1 release by chondrocytes was shown to have a migratory effect on CPCs, mediated via RAGE and toll-like receptor 4 (TLR-4). Using the collagenase-caused enzymatic injury properly, short-term enzymatic treatment could help to loosen cartilage tissue and accelerate CPCs migration into injured cartilage, differentiating into chondrocyte-like cells [36]. In subsequent studies, many other factors released after cartilage injury could also induce the migration of CPCs to the injury site, including stromal cell-derived factor 1 (SDF-1) [37], insulin-like growth factor 1 (IGF-1), and platelet-derived growth factor (PDGF) [38], suggesting that the migration was likely to be regulated by multiple injury-/inflammatory-related signaling pathways.

Danger/damage-associated molecular patterns (DAMPs) not only triggered the chemotaxis, but also elicited the immunomodulatory response of CPCs, showing a significant increase in pro- and anti-inflammatory gene expression [39]. Moreover, compared to chondrocytes, CPCs were more sensitive in their response to endogenous DAMPs which were released from dead cells, represented by the systemic inflammation mediator HMGB1 (a nuclear DAMP) and mitochondrial DAMPs (MTDs), with a higher upregulation of MMP-13, IL-6, and IL-8 in the latter group [40]. Moreover, CPCs also played a macrophage-like role in injured cartilage. Compared to chondrocytes, CPCs showed significantly higher rates of fluorophore-labeled fibronectin fragment engulfment, greater lysosome activity and mass, over-expressed phagocytosis-related genes and proteins, and increased cathepsin B-dependent clearance of cell debris [41].

#### 3.1.2. Migrating CPCs in Osteoarthritis

With the increased frequency and MSC surface markers in osteoarthritic cartilage, CPCs are recognized to be involved in the pathogenesis of osteoarthritis (OA) [27]. CPCs were reported to be mainly distributed on the surface of articular cartilage. When cartilage was damaged, CPCs tended to migrate to the injury site, as they did in the case of OA. However, the migration of CPCs was significantly inhibited by OA-associated cytokines, such as Interleukin-1 beta (IL-1β) and tumor necrosis factor alpha (TNF-α), contributing to the low regenerative potential of cartilage in vivo [38]. Additionally, Xia et al. revealed that in the instance of downregulated MiR-31-5p and miR-424-5p, the reduced percentage and proliferation ability and the increased osteogenic potential of CPCs contributed to the homeostasis imbalance in OA cartilage [28]. Although the migration was inhibited, the distribution and activation of CPCs were also observed in the middle-deep layers of OA articular cartilage, suggesting that the maldistribution of CPCs was closely related to the progression of OA [42,43].

In the OA articular cartilage, the phenotype of CSPCs correlating with the degree of ECM degeneration is likely to be different at different stages of OA. Tong et al. disclosed that CPCs were activated and exhibited a transient proliferative response in early OA as an initial attempt for self-repair, gradually quieting as the OA process was mediated by IL-1β via the NF-κB pathway [44]. In the study by Mantripragada et al. [45], although OA grade 3–4 cartilage showed greater cell concentration than did grade 1–2 cartilage, it harbored fewer CPCs. Moreover, compared to grade 1–2 CPCs, grade 3–4 CPCs expressed a higher CD271 and exhibited enhanced osteo-adipogenic activities, decreased chondrogenic capacity, and stronger cell migration in response to OA synovial fluids [46]. In addition to migration, the aging of CPC is also closely related to the occurrence and development of OA. In the study of Jacob et al., OA-CPCs exhibited elevated reactive oxygen species (ROS) levels, along with a relatively high percentage of senescent cells compared to non-OA CPCs, mediated via the release of the pro-inflammatory cytokines, IL-6 and IL-8 [47]. Interestingly, Zhao et al. revealed the relationship between obesity and OA: high doses of leptin decreased the ability of the CPCs to migrate, inhibited their chondrogenic potential, increased their osteogenic potential, and induced cell cycle arrest and senescence by activating the p53/p21 pathway and inhibiting the Sirt1 pathway [48].

The migration of CPCs is undoubtedly an objective pathological manifestation. When we design a treatment plan, whether to promote the migration of CPCs to achieve better cartilage regeneration or to inhibit migration to restore disturbed tissue architecture is a vital question whose answer depends, at least in part, on the stage of OA. At present, the roles of CSPCs in the onset and progression of OA have not been clarified. More research and evidence are urgently needed to determine the related mechanisms and devise CPC-targeted therapies.

### 3.2. The Migratory CPCs and Cartilage Regeneration

The stronger migration ability is shared by CPCs obtained via different methods and is an essential characteristic that distinguishes CPCs from MSCs. Migratory CPCs (MCPCs), which are defined by the isolation method (method of migration) with outstanding migration capacity, are of particular interest for research. To determine what role they play in cartilage regeneration and to achieve better cartilage repair, many researchers have targeted MCPCs for study (Table 2).

MCPCs were first isolated and identified from human OA-articular cartilage by Keoling et al. [49]. Most of the subsequent studies were conducted in the context of OA and attempted to clarify the relationship between the progression of OA and the role of MCPCs. In the process of exploring the mechanisms, researchers hoped to find therapeutic targets for OA and reverse the stage of OA. In addition, OA-MCPCs were compared with MCPCs from rheumatoid arthritis cartilage (RA-CPCs) [54]. These cells shared basic characteristics of MCPCs, but still exhibited the following main differences: RA-MCPCs expressed IL-17 receptors and resulted in the upregulation of IL-6 and matrix metalloproteinase 3 (MMP-3) and exhibited a reduced chondrogenic potential, indicating that IL-17 blockade might be a new therapeutic option for RA [54].

Other researchers constructed cartilage injury models (blunt impact, enzyme) and isolated MCPCs that migrated after stimulation. In the study by Seol et al. [26], blunt impact injury caused local chondrocyte death and induced the homing of MCPCs via HMGB-1 and RAGE-mediated chemotaxis. There are also studies using the previously mentioned method of migration to obtain MCPCs and exploring the basic phenotype and functions of MCPCs in non-disease states. Moreover, some studies have focused on comparing CPCs with cartilage-derived cells (a mixture of CPCs and chondrocytes) and chondrocytes, which are derived from MCPCs-depleted cartilage (non-MCPCs) or isolated for not adhering to the fibronectin-coated plate. This comparison is interesting because, in past studies, cartilage-derived cells were often referred to as chondrocytes. Compared with non-MCPCs, MCPCs expressed significantly more baseline mRNAs of MMP-13, CXCL12, and IL-6 and were more sensitive than non-MCPCs in response to proinflammatory damage-associated molecular patterns (DAMPs), especially mitochondrial DAMPs (MTDs) [40]. Unfortunately, the study on nasal septal MCPCs is limited. Using the method of migration in the nasal field for the first time, Elsaeaaer et al. showed the characterizations of the nasal septal MCPCs and clarified their progenitor cell features and potential for nasal cartilage in situ regeneration [11].

## 4. CPCs-Based Cartilage Tissue Engineering Strategies

Based on CPCs, many researchers have constructed tissue-engineered cartilage using a variety of scaffold materials, conducting in vivo experiments (Table 2). With the help of growth factors or functional scaffolds, the researchers realized bionics in structure and function through their ingenious design.

In CPCs-based cartilage tissue engineering, natural hydrogel materials (including collagen [65,66,67], hyaluronic acid (HA) [67,68,69,70], fibrin [68,71,72,73], alginate [66,74], fibronectin [75], methacrylated gelatin (GelMA) [76,77,78,79,80,81], agarose [82], chondroitin sulfonate [67], silk fiber [83]), and synthetic materials (including polyglycolic acid (PGA) [84], poly-L lactic acid (PLLA) [20,21], polycaprolactone (PCL) [20,21,70,80], polyurethane (PU) [72], poly (vinyl alcohol) (PVA) [77], poly (3-hydroxybutyrate-co-3-hydroxyvalerate) (PHBV) [85,86], hydroxypropyl cellulose (HPC) [87], and poly (lactic-co-glycolic acid) (PLGA) [88]) were widely used, and their applicability has been verified. In addition, some studies used cell-laden hydrogels for bio-printing, achieving satisfactory cartilage regeneration results [76,77,78,79,80]. Other researchers designed the scaffold based on the structure of the joint, printed the layered scaffold, and seeded different seed cells on different layers of the scaffold (usually, the surface layer was planted with CPCs, and the middle and deep layers were planted with BMSCs), thus producing an integrated construction of bone and cartilage [70,76,78,81]. Moreover, Yu et al. used recombinant human stromal cell-derived factor 1α (rhSDF-1α) to improve the in situ recruitment of CPCs and achieved the functional repair of full-thickness bovine articular cartilage without adding seed cells.

However, there are few studies regarding tissue-engineered cartilage based on NCPCs, and most of the studies only focus on the characteristics or functions of NCPCs. At the same time, because of the difficulty of constructing a nasal septal defect model, there are currently no in vivo experiments devising tissue-engineered nasal septal cartilage based on CPCs. Shafiee et al. seeded NCPCs on aligned electrospun nanofibrous Poly (L-lactide) (PLLA)/Polycaprolactone (PCL) hybrid scaffolds, and the proliferation and chondrogenic differentiation of NCPCs were significantly enhanced compared to those obtained from samples seeded on random scaffolds [20]. In the later research, Shafiee et al. compared the four popular candidates for cartilage tissue engineering, including NCPCs, ACPCs, BMSCs, and ADSCs [21]. Seeded on the previous nanofibrous scaffolds, their self-renewal potential, immunophenotype, chromosomal stability, chondrogenic potential, and proliferation rate were compared, and the superiority of NCPCs was confirmed [21].

## 5. Strategies to Promote CPCs Migration and Chondrogenesis

**Figure 5:**
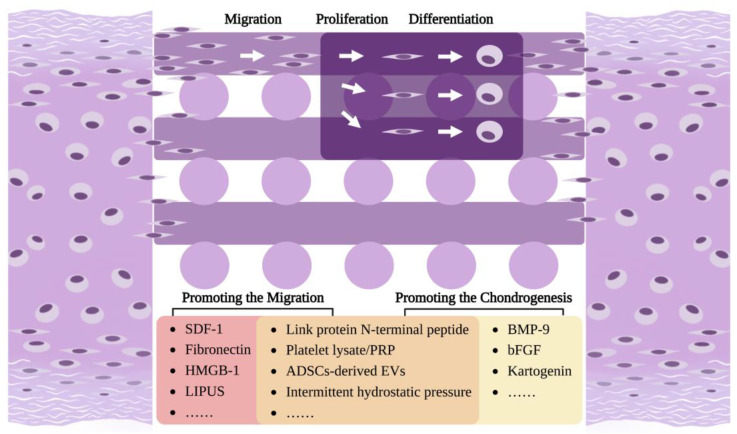
Strategies for promoting the migration and chondrogenesis of CPCs: After the tissue-engineered cartilage scaffold implantation into the cartilage defect, resident CPCs in the surrounding tissue are expected to undergo migration, proliferation, and differentiation to achieve the in situ regeneration of the cartilage. Some factors have been found to promote the migration of CPCs to the scaffold, and some other factors could promote the proliferation and chondrogenic differentiation of CPCs, while among these, some could promote both migration and chondrogenesis. Created with BioRender.com (accessed on 15 December 2022).

In order to achieve in situ regeneration, the migration of cells around the defective tissue is the most important link. CPCs have a strong ability for basic migration, and could respond to inflammatory factors and migrate to the injured site, which was fully discussed in the previous section. However, despite possessing resident progenitors committed to chondrogenesis, cartilage does not show strong intrinsic self-repair ability, indicating that migration and regeneration of the natural physiological/pathological levels is insufficient to repair cartilage defects [42]. Therefore, it is necessary for us to use scaffolds or factors (biological, chemical or physical) to induce more CPCs to migrate to tissue engineering scaffolds and then differentiate in order to promote the proliferation of chondrocytes in the target site and achieve cartilage in situ regeneration (Figure 5).

### 5.1. Promoting the Migration of CPCs

Stromal cell-derived factor 1 (SDF-1) is a key chemokine regulating stem cell migration and homing to sites of tissue damage through binding to the cell surface receptor CXCR4. In the study by Jayasuriya et al. [89], treating CPCs with CXCR4 blocker AMD3100 disrupted cell homing to injured sites and prevented fibrocartilage regeneration in meniscus tissue. In this way, SDF-1 has become a promising molecule for in situ cartilage regeneration by recruiting endogenous CPCs. Using a recombinant human SDF-1α-loaded fibrin/HA scaffold, Yu et al. achieved the functional repair of full-thickness articular cartilage via the homing of CPCs in bovine femoral condyle osteochondral explants [68]. Moreover, hydroxypropyl cellulose (HPC) scaffolds pretreated with SDF-1 stimulated CPCs migration and meniscal fibrocartilage repair in human explant tissue [87]. Fibronectin (FN) is a dimeric glycoprotein that exists in the plasma and ECM of various tissues. As the ligand of integrin α5β1, FN provides the molecule binding sites and participates in important processes including cellular migration, adhesion, and differentiation. With high expression levels of the integrin α5β1 (CD54 and CD29) [11,26,38,54], CPCs are promising for responding positively to FN and achieving ideal recruitment to the target site. In the study by Kalkreuth et al. [90], human plasma-derived FN stimulated the migration and proliferation of human subchondral progenitor cells. Additionally, Tao et al. demonstrated that FN could enhance cartilage repair by activating CPCs through the integrin α5β1 receptor in the knee of early-stage OA mouse models [75]. High mobility group box 1 protein (HMGB-1) is a protein primarily located in the nucleus, and it can be translocated to the cytoplasm and secreted during cell activation and cell death. In the study by Seol et al. [26], MCPCs were more active than CCs in chemotaxis assays and responded to HMGB-1. In the exploration of OA mechanism, Wagner et al. found that HMGB1 could be released by OA-CCs and exhibited migratory effects on CPCs via its receptors toll-like receptor 4 (TLR4) and the receptor for advanced glycation end products (RAGE) [35]. Moreover, in the study by He et al. [91], link protein N-terminal peptide (LPP) could stimulate the proliferation, site-directional migration, and chondrogenic differentiation of CPCs.

Natural combinations of growth factors, including platelet lysate (PL), platelet-rich plasma (PRP), and extracellular vesicles (EVs), could also promote CPCs migration, and at the same time, they have a prominent effect of promoting the chondrogenesis of CPCs. The platelet membrane is always ruptured in the PRP suspension to obtain the PL [62]. As products based on platelets, both possess a high content of platelet-derived growth factors and bioactive proteins, making them promising clinical therapeutic agents to restore damaged cartilage [92]. In the study by Carluccio et al. [62], CPCs recruited by PL were able to migrate in response to inflammatory stimuli, showed paracrine activity in attracting other cells to the injured sites, and displayed high chondrogenic potential and resistance to hypertrophy. In the PRP-based scaffold, the proliferation and chondrogenesis in CPCs were stimulated, and were stronger in CPCs than in BMSCs and chondrocytes [60]. EVs are membrane-bound nanovesicles, ranging in size from 30 to 500 nm, and containing bioactive molecules that migrate throughout the local microenvironment, exerting their effects on target cells and inducing endogenous repair mechanisms [93]. It has been proved that progenitor cells could activate resident cells to mediate tissue regeneration through paracrine mechanisms, and CPCs-derived EVs might play a comparable role in CPCs-mediated cartilage regeneration [94]. Moreover, ADSCs/BMSCs-derived EVs could promote the migration, proliferation, and chondrogenic differentiation of CPCs for cartilage tissue engineering as well [95,96,97]. Therefore, EVs from multiple sources could be used as effective additives.

In addition, some mechanical stimulation can also promote the migration of CPCs. Low-intensity pulsed ultrasound (LIPUS) promoted the migration of CPCs to the injured site via the focal adhesion kinase pathway [51]. Under the stimulation of intermittent hydrostatic pressure (IHP), the migration, proliferation and chondrogenic differentiation of CPCs were significantly promoted [74].

Similarly, the promotion of migration of chondrocytes, which had not yet been identified to be CPCs, was explored in some studies [98,99,100]. However, these cells migrated in response to the factors and showed characteristics similar to CPCs (especially MCPCs), appearing to play an important role in cartilage regeneration. Therefore, these studies may still be relevant when exploring the migration of CPCs.

### 5.2. Promoting the Chondrogenesis of CPCs

Compared to the promotion of chondrogenic progenitor cell migration, the promotion of chondrogenesis seems to be easier, as the previous systems of research on the induction of chondrogenic differentiation of stem cells and the promotion of chondrocyte secretion of cartilage matrix are more fully developed. In the study by Padmaja et al. [101], bone morphogenic protein 9 (BMP-9) could enhance the chondrogenic potential of CPCs in expansion, without affecting their low hypertrophic tendency, and the presence of the parathyroid hormone during expansion was shown to mitigate hypertrophy. In the study of Shen et al. [102], basic fibroblast growth factor (bFGF) could promote the proliferation and chondrogenesis of CPCs and inhibit osteogenesis, while a culture system containing 2 ng/mL bFGF was optimal. Kartogenin (KGN), a popular small molecule in cartilage regeneration, could repair full-thickness cartilage defects [103], and the effect of cartilage regeneration was partially stimulated by the IL-6/Stat3-dependent proliferation of CPCs [104]. Moreover, some of the factors promoting the chondrogenesis of CPCs have been described in the previous introduction of the promotion of migration. Moreover, some modifications of the scaffold material can also improve cartilage regeneration, and the related studies are summarized in Table 3.

In addition to factors that have been shown to promote CPCs chondrogenesis, other factors that could promote the chondrogenic differentiation of BMSCs and the proliferation of chondrocytes may also be considered as potentially effective additions [94,96,97,99,105,106]. However, while research ideas are provided, further research is needed to prove their efficacy and effectiveness.

**Table 3:** Studies on tissue-engineered cartilage based on CPCs and studies on tissue-engineered cartilage based on cartilage progenitor cells (CPCs). ACPCs, articular CPCs; NCPCs, nasal CPCs; BMSCs, bone marrow-derived mesenchymal stem cells; PLLA, poly-L lactic acid; PCL, polycaprolactone; HA, hyaluronic acid; PU, polyurethane; GelMA, methacrylated gelatin; PVA-MA, methacrylated poly(vinyl alcohol); HAMA, methacrylated HA; PHBV, poly (3-hydroxybutyrate-co-3-hydroxyvalerate); PRP, platelet-rich plasma; HPC, hydroxypropyl cellulose; MEW, melt electrowriting; PLGA, poly (lactic-co-glycolic acid); OA, osteoarthritis; rhSDF, recombinant human stromal cell-derived factor; BMP, bone morphogenetic protein; ADSCs, adipose-derived stem cells; FPSCs, infrapatellar fat pad-derived stem cells; ACs, articular chondrocytes; DLP, digital light processing; SDF, stromal cell-derived factor.

No.	Reference	Seed Cell	Scaffold	Implanted Site	Result
1	Williams et al., 2010 [65]	Human/Goat ACPC	Type I/III collagen membrane	Goat lateral femoral condyle	The ACPCs group and the chondrocytes group showed comparable histological repair scores.
2	Shafiee et al., 2014 [20]	Human NCPC	Nanofibrous PLLA-PCL	—	The chondrogenic differentiation of NCPCs was enhanced when cultured on aligned scaffolds compared with randomly oriented scaffolds.
3	Marcus et al., 2014 [107]	Bovine ACPC	—	SCID mouse thigh muscle	The ACPCs were able to survive in vivo, but failed to create a robust cartilage pellet, suggesting the requirement of further signals for chondrogenic differentiation.
4	Frisbie et al., 2015 [71]	Equine ACPC	Fibrin	Equine femur medial trochlear ridge	Autologous cells significantly reduced central osteophyte formation compared with allogenic cells and fibrin alone.
5	Yu et al., 2015 [68]	—	HA-fibrin	Bovine femoral condyle osteochondral explants	The use of rhSDF-1α improved ACPC recruitment and achieved functional repair of full-thickness bovine articular cartilage.
6	Neumann et al., 2015 [72]	Human ACPC	Fibrin + PU	—	Mechanical stimulation induced chondrogenesis; over-expression of BMP-2 increased hypertrophy markers.
7	Shafiee et al., 2015 [21]	Human NCPC/ACPC	Nanofibrous PLLA-PCL	—	NCPCs showed a higher proliferation potential and chondrogenic capacity than did BMSCs, ADSCs, and ACPCs.
8	Li et al., 2016 [74]	Rabbit ACPC	Alginate	—	Intermittent hydrostatic pressure enhanced the migration and chondrogenic differentiation of ACPCs, which were more prominent than in FPSCs and ACs.
9	Jiang et al., 2016 [73]	Human ACPC	Fibrin	Immunodeficient mouse back; human femoral condyle	2DLL-cultured ACPCs proved efficient in cartilage formation, both in vitro and in vivo, and in repairing large knee cartilage defects (6–13 cm^2^) in patients.
10	Studer et al., 2016 [66]	Human ACPC	Alginate + porous porcine collagen I/III sponge	Nude mouse subcutaneous pocket	Human ACPCs in alginate in collagen hybrid scaffolds produced stable cartilage in vivo.
11	Levato et al., 2017 [76]	Equine ACPC Equine BMSC	GelMA	—	Combining ACPC-laden and BMSC-laden bioink, articular cartilage consisting of defined superficial and deep regions was generated.
12	Tao et al., 2018 [75]	Mouse ACPC	Fibronectin/Pluronic F-127	Mouse knee (OA)	Fibronectin enhanced ACPCs proliferation, migration, and chondrogenic differentiation through the integrin α5β1-dependent signaling pathway.
13	Lim et al., 2018 [77]	Equine ACPC	Bio-resin (PVA-MA/GelMA)	—	DLP-printed bio-resin supported the chondrogenic differentiation of ACPCs.
14	Mouser et al., 2018 [78]	Equine ACPC Equine BMSC	GelMA/gellan/HAMA	—	The incorporation of HAMA improved shape-fidelity; chondrogenic differentiation was confirmed, while printing influenced this response.
15	Xue et al., 2019 [85]	Swine ACPC	PHBV	Nude mouse subcutaneous pocket	CPCs underwent chondrogenesis without chondrogenic induction and were better at performing chondrogenesis than were BMSCs, but worse than ACs.
16	Wang et al., 2019 [60]	Human ACPC	PRP	Rabbit knee	ACPCs exhibited superiority over BMSCs and ACs in PRP-based scaffold for cartilage regeneration.
17	Bernal et al., 2019 [79]	Equine ACPC	GelMA-based resin	—	ACPCs synthesized teh neo-fibrocartilage matrix in volumetric rapidly bioprinted meniscus-shaped constructs.
18	Newberry et al., 2019 [87]	Human CPC cell line	HPC	Human explant meniscus tissue	CPCs migrated in response to SDF-1 and successfully dispersed into injured tissues to help facilitate tissue reintegration.
19	Mancini et al., 2020 [70]	Equine ACPC Equine BMSC	HA/poly(glycidol)-based hydrogel + PCL	Equine knee	The repair tissue was significantly stiffer in defects repaired with ACPC/BMSC zonal constructs.
20	Peiffer et al., 2020 [80]	Equine ACPC	GelMA + MEW-PCL	—	The implant composed of ACPCs-laden hydrogel reinforced with an MEW scaffold retained its personalized shape, improved its compressive properties, and supported neocartilage formation.
21	Schmidt et al., 2020 [82]	Equine ACPC	Agarose	—	Higher production of glycosaminoglycans, weaker type I collagen staining, and lower alkaline phosphatase activity were observed in the ACPC constructs compared to BMSC constructs.
22	Bauza et al., 2020 [67]	Human ACPC	Collagen-chondroitin sulfate	—	OA-ACPCs with immunosuppressive potential had a higher proliferation rate and a higher propensity toward chondrogenesis compared to BMSCs.
23	Piluso et al., 2020 [83]	Equine ACPC	Silk fibroin	—	Rapid gelation of silk fibroin could be achieved by combining it with riboflavin and electron acceptors, while the contained ACPCs maintained their viability.
24	Wang et al., 2021 [88]	Human ACPC	PLGA	Rabbit knee	ACPC-loaded PLGA scaffolds produced a hyaline-like cartilaginous tissue, which showed good integration with the host tissue and subchondral bone.
25	Xue et al., 2022 [86]	Swine ACPC	PHBV-Bioglass	Nude mouse back subcutaneous tissue	The addition of bioglass improved the cell adherence, cartilage matrix formation, and biomechanical performance.

## 6. Summary and Future Outlook

Increasing evidence has shown that CPCs are promising seed cells for cartilage tissue engineering and have superior migration ability and in situ distribution compared to other seed cells, which have been fully discussed in the previous section. Therefore, the use of CPCs is expected to achieve in situ regeneration, which has great potential for clinical application and brings hope for the treatment of cartilage injury-related diseases. Currently, studies on articular cartilage progenitor cells are more comprehensive, while studies on nasal CPCs are yet to be further developed. The easier availability and larger surface layer in which CPCs are mainly distributed both illustrate the advantages of using NCPCs for in situ regeneration of nasal cartilage compared to those using articular cartilage. Moreover, with perichondrium on the surface, it is not clear whether perichondrium-derived progenitor cells or superficial cartilage-derived progenitor cells are the main force in nasal cartilage regeneration. Moreover, given that CPCs from the deep zone of articular cartilage possess greater chondrogenic and osteogenic potential [33], the differences of CPCs from different zones of nasal septal cartilage (superficial versus medial; posterior versus anterior) and different nasal cartilage (lobular versus septum) are worth exploring.

At the same time, nasal septal cartilage is often used as a source of cartilage autograft due to its easy accessibility and superior mechanical properties. Therefore, if regeneration of nasal septal cartilage can be achieved, it can not only solve the problem of the nasal septal defect, but also serve as a regenerable cartilage donor area, indirectly solving the problem of insufficient cartilage sources faced when diseases in other parts of the body require treatment with cartilage grafts, such as contracted nose deformity, cleft lip nasal deformity, microtia, osteoarthritis, etc.

However, studies on NCPCs and even CPCs are still insufficient in terms of the overall perspective. The study of different functional subpopulations of CPCs is still at a preliminary stage, and the mechanisms of the migration and participation of CPCs in tissue repair and the interaction and transformation of CPCs with mature chondrocytes are still unclear. More systematic and in-depth studies need to be completed to further elucidate the mechanisms involved, with subsequent improvement of the cartilage scaffold (modified polymer/hydrogel/biological scaffold) to increase its ability to recruit CPCs. The unresolved status of these issues not only affects the judgment of CPCs-based strategy security, but also limits the development of new strategies. Moreover, as one of the most important aspects of achieving in situ cartilage regeneration, the efficacy of CPCs recruitment requires further improvement. New strategies for improving the cartilage scaffold (modified polymer/hydrogel/biological scaffold), as well as new additives, are constantly emerging. However, none of these has yet showed absolute superiority. In addition, whether these strategies have other side effects is also an important issue to be determined. Additional in vivo and in situ animal experiments need to be completed, which would lay a foundation for clinical application and ultimately provide help for patients with nasal cartilage defects in the future.

## Figures and Tables

**Table 1:** Studies on human NCPCs and their isolation/culture methods and characteristics. ACPCs, articular cartilage progenitor cells; BMSCs, bone marrow-derived mesenchymal stem cells; ADSCs, adipose-derived stem cells; PDGF, platelet-derived growth factor; MMP, matrix metalloproteinase; CPCs, cartilage progenitor cells.

No.	Reference	Isolation	Culture	Characteristics and Results
1	Shafiee et al., 2011 [10]	Collagenase type I and II (0.2%, 16 h); Colony culture	DMEM-low glucose, 15% FBS, 10 μg/mL ascorbic acid, 1% penicillin-streptomycin solution, 1.25 μg/mL amphotericin-B	(+): CD90, CD105, CD106, CD133, CD166, HLA-ABC, S100, P75, GFAP (−): CD34, CD45, HLA-DR Multilineage differentiation capacity: chondrocytes, osteocytes, and neural-like cell types; without adipogenic differentiation potential.
2	do Amaral et al., 2011 [15]	Collagenase type I	α-MEM, 10% FBS, 100 U/mL penicillin, 100 μg/mL streptomycin	(+): CD105, CD73, CD44 (−): CD146 Multilineage differentiation capacity: Achieved chondrogenic differentiation without the addition of growth factors; Negative for the osteogenic master gene (CBFA-1), but deposit considerable amounts of extracellular calcium under osteogenic-inducing medium; Negative for the adipogenic master gene (PPAR-γ2); failed to differentiate into adipocytes and accumulate cytoplasmic lipid droplets.
3	Baptista et al., 2013 [6]	Collagenase type I	α-MEM, 20% FBS, 100 U/mL penicillin, 100 μg/mL streptomycin	Satisfactory cartilage formation in growth-factor-free 2D medium and pellet 3D culture.
4	Shafiee et al., 2014 [20]	Collagenase type I and II; Colony culture	DMEM-low glucose, 10% FBS, 100 U/mL penicillin, 0.1 mg/mL streptomycin	(+): CD73, CD90, CD105, CD106, CD166, HLA-ABC (−): CD34, CD45, CD133, HLA-DR Multilineage differentiation capacity: chondrocytes, osteocytes; without adipogenic differentiation potential; Ploy (L-lactide) (PLLA)/Polycaprolactone (PCL) nanofibrous scaffolds fabricated via electrospinning maintained NCPCs proliferation and differentiation, and the aligned scaffolds could significantly enhance chondrogenic differentiation.
5	Shafiee et al., 2015 [21]	Collagenase type I and II; Colony culture	DMEM-low glucose, 10% FBS, 100 U/mL penicillin, 0.1 mg/mL streptomycin	(+): CD73, CD90, CD105, CD106, CD133, CD166 (−): CD34, CD45, HLA-DR Retained a normal karyotype, and no chromosomal abnormalities were envisaged during long-term culture (passage 10). Higher chondrogenic potential, proliferation rate, and level of ECM production compared to ACPCs, BMSCs, and ADSCs.
6	Elsaesser et al., 2016 [11]	Migration	DMEM/Ham’s F12 (1:1), 10% FBS, 1% PS	(+): CD9, CD29, CD44, CD49d, CD49e, CD49f, CD54, CD 73, CD90, CD105, CD106, CD146, CD166 (−): CD31, CD34, CD45, CD133/1, CD133/2 Higher basal migratory activity than BMSCs and chondrocytes and could be significantly stimulated by PDGF-BB. Multilineage differentiation capacity: adipogenesis, osteogenesis and chondrogenesis. Stronger MMP-9 activation than chondrocytes.
7	Stuart et al., 2017 [22]	Collagenase type I	DMEM-low glucose, 10% FBS, 100 U/mL penicillin, 100 μg/mL streptomycin	(+): CD44 Formed CPC spheroids by micromolded nonadhesive hydrogel and achieved scaffold-free cartilage engineering without chondrogenic stimulus.
8	Kim et al., 2018 [23]	Collagenase; Colony culture	DMEM-low glucose, 10% FBS	(+): CD44, CD73, CD90, CD105, CD106, CD166, HLA-ABC (−): CD14, C19, CD34, HLA-DR Multilineage differentiation capacity: adipogenesis, osteogenesis, and chondrogenesis. The characteristics were not influenced by prolonged cultivation (Passage 10).
9	Jessop et al., 2020 [24]	Fibronectin adhesion assay	DMEM, 10% FBS, 1% PS, 1 mM d-glucose, 0.1% MEM-non-essential amino acids	(+): CD29, CD44, CD56, CD73, CD90 (−): CD34, CD45 Multilineage differentiation capacity: adipogenesis, osteogenesis, and chondrogenesis. Increased expression of CCND1, CCND2, NCAM1, and CDH2 compared to differentiated chondrocytes. Maintains the phenotypic stability of chondrocytes through influence on dedifferentiation.

**Table 2:** Studies on MCPCs and their roles in cartilage regeneration. OA, osteoarthritis; RA, rheumatoid arthritis; CPCs, cartilage progenitor cells; HMGB, high mobility group box; RAGE, receptor for advanced glycation end products; PDGF, platelet-derived growth factor; IGF, insulin-like growth factor; IL, interleukin; TNF, tumor necrosis factor; CCL, chemokine (C-C motif) ligand; IP3R, inositol 1,2,3-trisphosphate receptor; STIM, stromal interacting molecule; ORAI, ORAI calcium release-activated calcium modulator; rhSDF, recombinant human stromal cell-derived factor; BMSCs, bone marrow-derived mesenchymal stem cells; ECM, extracellular matrix; VEGF, vascular endothelial growth factor; SDF, stromal cell-derived factor; CXCR, C-X-C chemokine receptor; HIF, hypoxia inducible factor; EGF, epidermal growth factor; TLR, toll-like receptor; PRP, platelet-rich plasma; SMURF, SMAD specific E3 ubiquitin protein ligase; DAMPs, danger/damage-associated molecular patterns; MTDs, mitochondrial DAMPs.

No.	Reference	Species	Background	Results
1	Koelling et al., 2009 [49]	Human knee joint	OA	CPCs with high migratory and chondrogenic potential were harbored in the late-stage OA articular cartilage.
2	Koelling et al., 2010 [50]	Human knee joint	OA	Concentrations of testosterone and estrogen influenced the expression of receptor genes and had a positive effect on the chondrogenic potential of CPCs by regulating the gene expression of Sox9, Runx2, type II collagen, and type I collagen.
3	Seol et al., 2012 [26]	Bovine stifle joint and human knee joint	Healthy/ Non-OA	Blunt impact injury caused local chondrocyte death and induced homing of CPCs via HMGB-1 and RAGE-mediated chemotaxis.
4	Joos et al., 2013 [38]	Human knee joint	OA	Traumatized cartilage released chemoattractive factors like PDGF-BB and IGF-1 for CPCs, but IL-1β and TNF-α inhibited their migratory activity, which might contribute to the low regenerative potential of cartilage in vivo.
5	Jang et al., 2014 [51]	Bovine stifle joint	Healthy	Low-intensity pulsed ultrasound stimulated the migration of CPCs toward the injured area of cartilage through focal adhesion kinase activation.
6	Zhou et al., 2014 [52]	Bovine stifle joint	Healthy	CPCs overexpressed chemokines IL-8 and CCL2 and were phenotypically more similar to synoviocytes and synovial fluid-derived cells than chondrocytes.
7	Matta et al., 2015 [53]	Human knee joint	OA	CPCs expressed IP3R, STIMI, and Orai1 proteins and were negative for RYR, and Ca2+ signaling played a role in CPCs differentiation.
8	Yu et al., 2015 [37]	Bovine stifle joint	Healthy	The use of rhSDF-1α improved the recruitment of CPCs and achieved functional repair of full-thickness articular cartilage.
9	Jiang et al., 2015 [37]	Human knee joint	OA	CPCs were activated in OA via interleukin-1β/nerve growth factor signaling.
10	Schminke et al., 2015 [54]	Human knee joint	OA and RA	IL-17 upregulated RUNX2, IL-6 and MMP-3, reducing the chondrogenic potential of RA-CPCs, while antagonizing IL-17 activity could enhance the anti-inflammatory IL-10 secretion and restore the chondrogenic potential.
11	Elsaeaaer et al., 2016 [11]	Human nasal septum	Healthy	CPCs showed higher migratory capacity compared to BMSCs and chondrocytes and similar ECM secretion to chondrocytes.
12	Zhou et al., 2016 [41]	Bovine stifle joint	Healthy	CPCs played a macrophage-like role regarding injured cartilage and showed time/cathepsin B-dependent clearance of cell debris, which was much more efficient than chondrocytes.
13	Seol et al., 2016 [55]	Bovine stifle joint-meniscus	Healthy	Injuries to the meniscus could mobilize CPCs with chondrogenic potential and the capacity for the repair of the cartilaginous white zone.
14	Wang et al., 2017 [56]	Bovine stifle joint	Healthy	The VEGF expression of CPCs could be stimulated by SDF-1 via p38 MAPK activation, which could self-sustain with the co-expression of SDF-1 and its receptor CXCR4.
15	Batschkus et al., 2017 [57]	Human knee joint	OA	The joing underwent qualitative and quantitative analysis of the secretome of CPCs by mass spectrometry.
16	Nguyen et al., 2018 [58]	Human knee joint	Non-OA	Platelet lysate induced the increase in HIF-1α, its nuclear relocation, and the binding to HIF-1 responsive elements, inducing quiescent cartilage cell activation and proliferation, leading to new cartilage formation.
17	Janssen et al., 2019 [59]	Human knee joint	OA	TGFβ3 and EGF stimulation influenced biglycan, SOX9, and RUNX2, while changes in the expression of their receptors contributed to degenerative/regenerative changes in late OA.
18	Wagner et al., 2019 [35]	Human knee joint	OA	HMBG1 released by chondrocytes had a migratory effect on CPCs, mediated via RAGE and TLR4.
19	Wang et al., 2019 [60]	Human knee joint	Non-OA	CPCs displayed superiority over BMSCs, chondrocytes, and PRP alone in PRP-mediated chondral defect regeneration.
20	Matta et al., 2019 [61]	Human knee joint	OA	A repository of quantitative proteomic data on the surfaceome of MSCs and CPCs relevant to cartilage biology and OA was established.
21	Wang et al., 2020 [46]	Human knee joint	OA	More OA grade 3–4 CPCs migrated to injured cartilage than did grade 1–2 CPCs, but with enhanced osteo-adipogenic and decreased chondrogenic capacity, which might explain the pathological changes in the CPCs during the progression of OA from early to late stages.
22	Carluccio et al., 2020 [62]	Human hip joint	—	Platelet lysate-recruited CPCs were able to migrate in response to inflammatory stimuli, showed paracrine activity in attracting other cells toward injured sites, and displayed high chondrogenic potential and resistance to hypertrophy.
23	Morgan et al., 2020 [63]	Bovine metacarpophalangeal joint	Healthy	As a potent chondrogenic factor for CPCs, BMP-9 was capable of inducing morphogenesis of adult-like cartilage, a highly desirable attribute for in vitro tissue-engineered cartilage.
24	Schminke et al., 2020 [64]	Human knee joint	OA	SMURF1 and SMURF2 were regulatory players for the expression of the major regulator transcription factors RUNX2 and SOX9 in CPCs from articular cartilage and meniscus.
25	Ding et al., 2021 [40]	Bovine stifle joint	Healthy	Compared with non-CPCs, CPCs expressed significantly more baseline mRNAs of MMP-13, CXCL12, and IL-6, and were more sensitive than non-CPCs in response to DAMPs, especially MTDs.
26	Vinod et al., 2021 [34]	Human knee joint	OA	Migratory CPCs expressed higher levels of CD146 and CD49b and retained superior intrinsic chondrogenic potential as compared to fibronectin-derived CPCs.

## Data Availability

Not applicable.
